# Fragmentation of Healthcare Services as a Possible Determinant of the Low Completion for the Tuberculosis Cascade of Prevention among Asylum Seekers: Results from a Prospective Study with Historical Comparison

**DOI:** 10.3390/pathogens11060613

**Published:** 2022-05-24

**Authors:** Valentina Marchese, Paola Zanotti, Claudia Cimaglia, Benedetta Rossi, Beatrice Formenti, Paola Magro, Maurizio Gulletta, Giovanna Stancanelli, Issa El-Hamad, Enrico Girardi, Daniela Maria Cirillo, Francesco Castelli, Alberto Matteelli

**Affiliations:** 1 Department of Infectious and Tropical Diseases, University of Brescia and ASST Spedali Civili of Brescia, 25123 Brescia, Italy; paolazanotti87@gmail.com (P.Z.); benedetta.rossi19@gmail.com (B.R.); beatriceformenti1992@gmail.com (B.F.); magropao@gmail.com (P.M.); mauriziogulletta@yahoo.it (M.G.); issa1957@libero.it (I.E.-H.); francesco.castelli@unibs.it (F.C.); alberto.matteelli@unibs.it (A.M.); 2 WHO Collaborating Centre for TB Elimination and TB/HIV Co-Infection, Department of Clinical and Experimental Sciences, University of Brescia, 25123 Brescia, Italy; 3 Clinical Epidemiology Unit, Lazzaro Spallanzani National Institute for Infectious Diseases, 00149 Rome, Italy; claudia.cimaglia@inmi.it (C.C.); enrico.girardi@inmi.it (E.G.); 4 Emerging Bacterial Pathogens Unit, IRCCS San Raffaele Scientific Institute, 20132 Milan, Italy; giovanna.stancanelli@gmail.com (G.S.); cirillo.daniela@hsr.it (D.M.C.); 5 UNESCO Chair “Training and Empowering Human Resources for Health Development in Resource-Limited Countries”, Department of Clinical and Experimental Sciences, University of Brescia, 25123 Brescia, Italy

**Keywords:** tuberculosis, migrant, Europe, TB infection, LTBI, healthcare delivery

## Abstract

**Background:** Effective screening for tuberculosis infection (TBI) among asylum seekers (AS) is crucial for tuberculosis (TB) elimination in low incidence countries. **Methods:** We assessed the proportion of completion of the screening for TBI among asylum seekers with a centralized delivery method compared to the decentralized model previously adopted in the study area (historical control). In the historical model (January 2017 to May 2018) screening of AS was performed at the arrival offering TBI testing (TST followed by IGRA among those positive), radiological investigation, treatment initiation and hospital referral, if needed, at three sites: migrant health clinic, pneumology clinic and infectious diseases department for active disease (decentralized model). In the study model (June 2018, centralized) all steps of screening were performed at a single site, at a minimum of 6 months after arrival. Multivariable Poisson regression analysis, with robust variance, was used to assess variables associated with the completion of screening for infection. Multivariable logistic regression was used to identify factors associated with the diagnosis of TB infection. **Results:** The intervention approach was offered to 144 AS with an overall 98.6% proportion of completion (98.7% for those with a positive TST). In the historical screening model, 1192 AS were candidates for screening, which was completed by 74.5% of those who started it (44.7% for those resulted TST positive). Major losses (55%) were detected in the TST/CXR-IGRA sequential step, followed by the execution of TST test (25%). The ratio of screening completion was significantly higher in the intervention period (aIRR 1.78, 95% CI 1.68–1.88) and for AS coming from high incidence TB countries (aIRR 1.14, 95% CI 1.04–1.25). Screening after 6 months from arrival and age were associated with TB infection (2.09, 95% CI 1.36–3.2 and 1.14, 95% CI 1.01–1.29). **Conclusions:** Screening for TBI can be improved by a centralized approach. Higher prevalence of TBI 6 months after arrival could reflect recent (either during travel or in Italy) acquisition of the infection.

## 1. Introduction

Migration has been constantly increasing worldwide in recent decades [1]. Particularly since the late 2000s, the WHO European Region has experienced an influx of refugees and migrants, with a rapid increase occurring in 2015 [2]. Migrant pressure remained especially high in the Mediterranean area, although declining in Italy during the last 3 years [3].

Migration is associated with an increased risk of tuberculosis (TB) infection/disease, with higher rates of detection in many Western European Countries [4] and should be faced with tailored interventions to achieve TB elimination in these areas [5]. The risk of TB in migrants depends on several factors, such as the incidence of the disease in the country of origin, the higher HIV/TB coinfection prevalence, the type of migration journey and the socio-economic disadvantaged status in the host country [6]. Post-arrival reactivation of previous infection is one of the main drivers of TB among refugees and migrants [7].

Migrants often live and travel in crowded conditions, in an environment that can favors the transmission of tubercle bacilli or the progression from infection to active disease. Forced migrants, as asylum seekers (AS) and refugees, represent a special category with higher rates of active TB, due to the precarious living conditions in refugee camps and the interruption of healthcare services in the country of origin because of socio-political instability or war [8].

Nevertheless, screening and treatment completion ratios for tuberculosis infection (TBI) remain low [4], and novel strategies are required to improve both effectiveness and cost effectiveness.

Two systematic reviews were performed to assess effectiveness and cost effectiveness of screening for both active TB and TBI among migrants in EU/EEA [9,10]. For active TB screening, the highest yield and cost effectiveness was reached in programs targeting migrants from high-TB-incidence countries, although the best threshold of TB incidence still needs to be determined. Symptom-based screening has low sensitivity and should be limited to emergency settings, where a chest X-ray (CXR) cannot be performed or in case of a high influx of migrants (as at first arrival countries in Southern Europe in recent years, or in transit camps) [10]. For TBI, the effectiveness of testing and treatment programs are limited by the use of poorly predictive tests, long-length treatments and a weak care cascade [9]. Increasing screening uptake and treatment completion are required to provide individual and public health benefits. 

In European Union, the European Centre for Disease Control (ECDC) recommends screening all migrants coming from high-TB-incidence country for active TB with CXR, and for TBI, either using tuberculin skin test (TST) or interferon-gamma release assay (IGRA). However, certainty of evidence remains low and large heterogeneity of implementation still exists within countries [11]. 

Since 2016, the University of Brescia has been a partner of an international multicenter research project funded by the European Union’s Health Programme 2014–2020: E-DETECT TB project, “early detection and integrated management of tuberculosis in Europe” [12]. The project aims to implement TB prevention, diagnosis and treatment in six different European countries (Italy, Romania, Bulgaria, Sweden, United Kingdom and The Netherlands).

Within the project, we previously performed a retrospective analysis on screening for both active TB and TB infection among AS in Brescia in 2015–2016 [13]. We detected high TB prevalence and incidence and a poor performance of the symptom-based screening. The cascade of care for prevention was affected by significant losses: screening for infection was completed by 49.0% of the eligible. Among those who completed the screening, 47.9% initiated the treatment, which was completed by 63.6% of them eventually [13]. The complex and fragmented organization of health services was identified as a possible determinant of the poor performance of the cascade.

Here, we report, in the same population and setting, the effect of a centralized “one-stop-shop” service organization on the ratio of screening completion.

## 2. Methods

### 2.1. Setting and Study Population

We conducted a prospective cohort analysis of the cascade of secondary prevention for TB in AS in the province of Brescia, Northern Italy, an urban area with a population of about 1,200,000 inhabitants. At the beginning of 2021, the city registered 19.1% of foreign-born residents, one of the higher urban percentages in Italy after Prato (25.3%) and Milan (20.1%). The whole province registered 12.4% which is still higher than national average percentage (8.5%) [14].

The study population consisted of the AS arriving at the second level reception centers (those hosting AS after the first arrival on borders while the request for asylum visa is processed). In June 2018, a novel centralized model was implemented for all AS who were in the study area for at least 6 months and had not yet been screened for TBI. Historical comparison (from January 2017 to May 2018) was used to assess the effectiveness of the novel strategy. The ratio of screening completion was compared between the study and control periods. 

### 2.2. Screening Procedures 

According to local health policies, all new migrants were offered a voluntary and free-of-charge screening for active TB and TBI [15].

At the first medical evaluation, all AS were investigated for TB through a clinical examination and a standardized verbal questionnaire to look for any signs and symptoms suggestive of TB, including persistent fever (more than one week), cough lasting ≥ two-weeks, night sweats, weight loss and haemoptysis. Screening took place in a first level structure, the Migrant Health Clinic, or in the hosting center using a mobile unit, depending on the recipient’s preference. Screen-positive individuals were referred to a second level structure, the ASST Spedali Civili Hospital, where a CXR was systematically performed. In case of radiological abnormalities, further investigations included at least microscopic examination, Xpert MTB-RIF and culture of the sputum. TB cases were then managed in the same second level structure until treatment completion. 

#### 2.2.1. Centralized Screening for TBI (June 2018)

The centralized procedure was implemented in line with updated regional policies [15]. According to the policy, to increase its effectiveness, screening for infection was delayed of six months after arrival. 

All the AS present in the area in June 2018 who were still unscreened for TBI and negative for active TB, were tested and treated using a centralized procedure, at the Infectious Diseases Department of Spedali Civili Hospital, were a dedicated multidisciplinary team was created. A TST was performed and on the same day of the TST reading, positive patients underwent an IGRA test and CXR. The team scheduled an appointment two weeks later for the discussion of results. Positivity of both tests without clinical and radiological signs of the disease defined TBI and indicated evaluation for treatment. 

#### 2.2.2. Decentralized Screening for TBI (January 2017 to May 2018)—Historical Control

Screen-negative individuals for active TB were addressed for testing for infection. As a first step, a tuberculin skin test (TST) was performed following the Mantoux technique, TST was considered positive with an induration of 10 mm or greater. Positive TST patients were prescribed IGRA test and CXR, to be performed in any available laboratory or clinic and followed by a specialist consultation at the Pneumology Outpatient Clinic of the ASST Spedali Civili of Brescia, where preventive therapy was offered if appropriate. 

### 2.3. Preventive Therapy

Three options were available for preventive therapy, the choice being a responsibility of the health care provider: rifampicin 600 mg plus isoniazid 300 mg per day for a duration of three months, rifampicin 600 mg daily for 4 months or isoniazid 300 mg daily for 6 months. Follow-up was performed monthly in the same structure through a medical visit and a blood test examination (blood cell count and transaminases). 

### 2.4. Data Analysis

Descriptive analysis of the characteristics of the two cohorts at baseline was performed. Median values and interquartile ranges (IQR) were used to describe numerical variables, while counts and percentages were employed for qualitative variables. Chi-square (χ^2^) test or Mann–Whitney non-parametric tests were used to compare groups for categorical or continuous variables, respectively.

We classified countries at high TB incidence according to WHO estimates for 2019 (threshold 150/100,000 population).

Asylum seekers were classified into high or low influx period according to the median number of arrivals per month during the study period, which was 35 migrants/month. Months with more than 35 arrivals were defined as “high influx” and the others were defined as “low”. 

TB prevalence was calculated as the ratio between the number of diagnosed TB cases occurring within 6 months from the arrival and the number of all asylum seekers/100,000. TB cumulative incidence was calculated as the ratio between the new TB cases diagnosed after 6 months from the arrival and the number of all asylum seekers at risk/100,000. 

The 95% confidence interval (95% CI) for TB prevalence and TB cumulative incidence were computed assuming a binomial distribution. Multivariable Poisson regression analysis, with robust variance, was used to assess variables associated with screening completion for infection, calculating incidence rate ratios (IRR) and their 95% CIs. Gender, age at arrival, TB incidence in the country of origin, influx period and screening procedures (centralized screening vs. decentralized) were included in the multivariable model. Multivariable logistic regression was used to identify factors associated with the diagnosis of TB infection, reporting the odds ratio (OR) and relative 95% CIs. The multivariable model included gender, age at arrival, TB incidence in the country of origin and screening timing (over 6 months from arrival in Italy vs. within 6 months from arrival).

All statistical analyses were performed using STATA release 15 (StataCorp. 2017. StataCorp LLC, College Station, TX, USA). A *p*-value less than 0.05 indicated conventional statistical significance.

### 2.5. Ethical Aspects

The study protocol received ethical approval from the Ethics Committee of the province of Brescia (study number 2901, date of approval 21 November 2017). All data were collected and analyzed according to current Italian laws for management of sensitive data. Standard clinical practice at all health care services included thorough information and verbal consent to any of the offered practices. Communication was facilitated by cultural mediators, as appropriate.

## 3. Results

### 3.1. Subjects Enrolled

Overall, from January 2017 to June 2018, 1356 AS were resettled in the province of Brescia. 

Most migrants came from Sub-Saharan Countries (1010 AS, 74.5%), namely Nigeria, (13.3%), Guinea (11.9%), Mali (9.0%), Gambia (8.9%) and Ivory Coast (8%). Asian migrants were 288 (21.2%) and came almost totally from Bangladesh (13.0%) and Pakistan (5.9%). Migrants were mostly from high-TB-incidence countries and arrived mainly during 2017 (73.6% in first semester and 21.5% during the second, respectively). Only 4.9% came in the first semester of 2018. Overall, 91.6% of migrants came during a high influx period.

Symptom-based screening for active TB tested positive in 57 subjects (4.2%). Nine TB cases were diagnosed and classified as prevalent (diagnosed within the first six months from the arrival). The susceptibility test revealed 1 multi-drug-resistant (MDR) TB. Estimated TB prevalence was 664/100,000 (95% CI 304–1256). Four additional TB cases were diagnosed at least six months after arrival and considered as incident cases. None of them presented symptoms at arrival, one had a negative IGRA following TST positivity and developed symptoms 12 months later. Three had positive TST and diagnosis was achieved following the detection of abnormalities at CXR during the screening for TB infection (performed in centralized, delayed procedure). Estimated TB cumulative incidence was 297/100,000 persons (95% CI 81–759).

For TBI screening, we excluded AS with active TB and those with symptoms for whom a CXR was not available (lost to follow-up). 

### 3.2. Screening Performance

During the intervention, 144 AS were screened and included in the study, compared to 1192 AS included in the control period. Table 1 summarizes the main demographic characteristics of the two cohorts. For the centralized procedure the median days of staying since the arrival in the study area to the execution of TST was 381 days (289–394), while it was 4 days (IQR 2–13) in the decentralized historical model. AS without a TST result or CXR/IGRA were lost to follow-up (2, 1.4% in the centralized model, 187, 20.9% in the historical control).

Age, sex and TB incidence in country of origin did not differ between the two cohorts. People screened with centralized procedure were most likely to come from Sub-Saharan countries compared to those in decentralized procedure and arrived less frequently during the high influx period. In both cohorts, all AS with a diagnosis of either active TB or TBI underwent HIV test, none of them were positive. 

**Figure 1:**
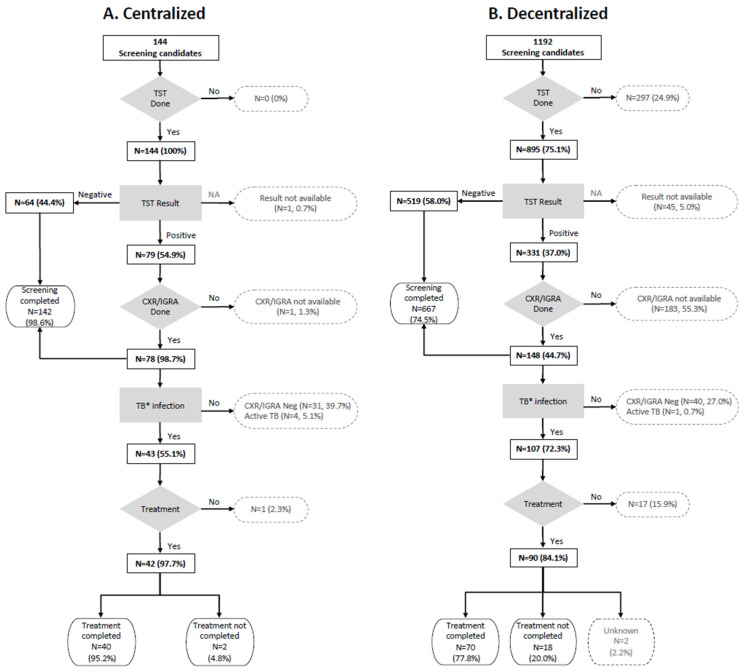
Flow-chart centralized procedure (panel (**A**)) and decentralized procedure (panel (**B**)). * positive TST and IGRA without radiological and clinical signs of disease.

Flow-charts reported in Figure 1 summarize infection screening results in centralized and decentralized procedures, respectively. TST reading was performed in 99.3% (143/144) in the centralized procedure and in 71.3% (850/1192) subjects in the decentralized one. CXR and IGRA tests were performed in 98.7% and 44.7%, respectively, of those who were TST positive. Screening completion ratio was 98.6% (142/144) in centralized screening and 55.9% (667/1192) in the decentralized procedure.

At the multivariable Poisson regression analysis, migrants screened by the centralized approach were significantly more likely to complete screening (aIRR 1.78, 95% CI 1.68–1.88, *p* < 0.001) as compared to those screened using the decentralized procedures. Similarly, AS coming from incidence countries with an incidence above 150/100,000 population (aIRR = 1.14; 95% CI: 1.04–1.24, *p* = 0.04) had a higher probability to complete the screening (Table 2). 

Of the 809 migrants who completed the screening (Table 3), TBI was diagnosed in 150 (18.5%). At multivariable logistic regression screening after 6 months from arrival (aOR = 2.09; 95% CI: 1.36–3.20, *p*-value = 0.001), and age (aOR = 1.14, 95% CI: 1.01–1.29, borderline statistically significant) were associated with TBI.

Treatment for TBI (six-month isoniazid in 94.4% cases) was prescribed to 90 of the 107 individuals (84.1%) in decentralized screening, while all treated patients received isoniazid-rifampicin therapy for 3 months in the centralized procedure (42/43, 97.7%). The latter procedure achieved a higher ratio of completion (77.8% vs. 95.2%). 

## 4. Discussion

The “one-stop-shop” service implemented in the centralized procedure achieved a better ratio of screening completion for TB infection in AS.

Intersectoral communication and service organization are essential to ensure adequate access to health services for AS [16]. Usually, non-governmental and voluntary organizations bridge gaps in the public health system by supporting examination booking, administrative procedures and cultural mediation. After the legal changes regarding the migrant reception system introduced at the end of 2018 [17], this support was reduced in Lombardy. Moreover, large numbers of AS may overwhelm the capacity of the staff in hosting centers. Booking and performing health consultations may be challenging (i.e., risk of overlapping the appointments, lack of vehicles for transportation), reducing the effectiveness of the screening. We speculate that the decentralized screening structure amplified bureaucracy. In our centralized intervention, we adopted simplified booking procedures (performed by the healthcare personnel of the dedicated multidisciplinary team) and a coordinated organization of consultations “per center”. This way, even a single social operator of the hosting center may provide assistance to different persons for which an appointment was booked on the same day. These strategies helped reduce the barriers to screening completion which are related to bureaucracy and logistics. In support of the major role played by logistics in the Italian setting, similar high losses in linkage from initial screening sites to diagnostic clinics were reported in an experience in Milan in the same period (2016–2017). In this study, Villa et al. identified a higher ratio of losses (41%) in screening completion rather than in the follow-up and treatment after diagnosis [18]. In the decentralized model, we found the highest dropout (55%) in the TST-IGRA/CXR step, the one with highest logistic and bureaucracy burden (i.e., booking procedures for blood tests, CRX and consultations at different sites, transport of AS to the clinics), also for the hosting center with dedicated personnel.

Barriers to accessing healthcare are well highlighted in the literature, such as the absence of cultural mediation, the lack of guidance on rights, cultural communication and expectations [19]. In our centralized experience, all steps of the screening were performed by the same multidisciplinary team, possibly contributing to an improved trusting in the system and a better relationship with the patients, both affecting the overall acceptance and completion of the screening. 

The six-month delay for the centralized screening could have selected a more “stable” population. However, this limited time of permanence is unlikely to have counteracted all barriers to healthcare access for AS. Indeed, the language barrier seems to not be reduced by a six-month stay, as suggested by a study performed in US, in which no changes were seen in the probability of speaking well by the fourth year since migration [20]. On the other hand, there are scarce data about the factors affecting the attitude to treatment initiation for TBI among migrants, but in one study performed in the Netherlands, the intended duration of stay (>5 years) was associated with higher treatment initiation [21]. 

The lack of qualitative data is a limitation of this study; however, the idea of Italy as a transit country is well known among AS coming through the Central Mediterranean route [22], and the six-month period seems too short to define the intention of permanent stay [23]. The small size of the cohort screened with the centralized procedure is another limitation of the study. A possible explanation for these results could be the limited number of arrivals in the second part of the study period (those in which the procedure has been mostly implemented), for which the European measures adopted in 2017 to reduce irregular immigration through the Central Mediterranean route would have possibly played a major role, as seen at the Italian borders [24,25].

The association of high TB incidence in the country of origin and odds of completing the screening is also interesting. In other studies, the attitude to initiate treatment has been associated with knowledge of relatives affected by TB [26], but there is scarcity of studies on the correlation between the ratio of screening completion and TB incidence in the country of origin. The higher incidence TB rate in the country of origin could contribute to the awareness of the disease and to have a better attitude to prevent it. However, more research is needed to identify gaps and strategies to improve access to preventive services among migrants, which remains low not only for TBI [27].

At the same time, the choice of administering mainly short regimens for TB infection in the centralized procedure largely influenced the higher treatment completion ratio observed, as previously documented and suggested in other settings [4,28].

The higher prevalence of infection found in migrants screened 6 months after their arrival is possibly due to recent infection, either acquired during the travel or in Italy. Similar considerations apply for the diagnosis of active TB performed in asymptomatic or TST-negative people at arrival. Other studies have highlighted the role of travel in infection transmission [6,7,29,30]. Although travel details were not collected in our study, the vast majority of AS entered Italy via the Central Mediterranean route, considered one of the most dangerous migration routes worldwide, for which overall precarious travel and living conditions are well documented [31].

An app-based health card and recording system were piloted within the project and found to be useful by healthcare professionals for tracing both active TB [32] and TBI and should be better investigated in larger studies. Improving an effective health record system and transfer, which is a transversal issue in migration health, both at national and international level, should be considered an essential tool for TB control and elimination in Europe, in order to guarantee both a continuum of care and completion of screening procedures [33], especially considering the recent finding from UK national data, in which IGRA-positive migrants without treatment had a 30 times higher risk of TB compared to TBI-negative migrants [34].

## 5. Conclusions

Simplification of the delivery care system for migrants is likely to play a major role in increasing the effectiveness of tuberculosis infection screening and treatment programs.

## Figures and Tables

**Table 1:** Demographic characteristics.

	Centralized Screening	Decentralized Screening	Total	*p*-Value
	N (%)	N (%)		
Total	144 (10.8)	1192 (89.2)	1336 (100)	
Gender				
Male	125 (86.8)	1035 (86.8)	1160 (86.8)	0.994
Female	19 (13.2)	157 (13.2)	176 (13.2)	
Age at arrival (years)				
≤20	66 (45.8)	544 (45.6)	610 (45.7)	0.964
>20	78 (54.2)	648 (54.4)	726 (54.3)	
Age (years), median (IQR)	22 (19–27)	22 (19–26)	22 (19–26)	0.342
Area of origin *				
Sub-Saharan Africa	134 (93.1)	857 (71.9)	991 (74.2)	<0.001
Other	10 (6.9)	335 (28.1)	345 (25.8)	
TB incidence in country of origin ^				
<150/100,0000	56 (38.9)	460 (38.6)	516 (38.6)	0.945
≥150/100,0000	88 (61.1)	732 (61.4)	820 (61.4)	
Influx period ^#^				
Low	28 (19.4)	82 (6.9)	110 (8.2)	<0.001
High	116 (80.6)	1110 (93.1)	1226 (91.8)	
Semester of arrival				
1st 2017	56 (38.9)	92 (77.9)	985 (73.7)	<0.001
2nd 2017	74 (51.4)	213 (17.9)	287 (21.5)	
1st 2018	14 (9.7)	50 (4.2)	64 (4.8)	

Abbreviation: IQR, Interquartile range. * Geographical regions used by the Statistics Division of the United Nations Secretariat (https://unstats.un.org/unsd/methodology/m49/, accessed on 1 April 2022). ^ Rates per 100,000 population. Global Tuberculosis Report—WHO 2020. ^#^ High influx periods: months with a number of arrivals greater than the median 35 migrants/month.

**Table 2:** Incidence rate ratio (IRR) and 95% confidence intervals (CI) for screening completion among asylum seekers.

	N. Screening Completed/Tot (809/1336)	aIRR (95% CI)	*p*-Value
Screening procedure			
Decentralized	667/1192	Ref.	
Centralized	142/144	1.78 (1.68–1.88)	<0.001
Gender			
Female	95/176	Ref.	
Male	714/1160	1.13 (0.98–1.29)	0.090
Age at arrival, by 5 years increase		1.00 (0.97–1.02)	0.741
TB incidence in country of origin			
<150/100,0000	287/516	Ref.	
≥150/100,0000	522/820	1.14 (1.04–1.25)	0.004
Influx period			
Low	67/110	Ref.	
High	742/1226	1.06 (0.92–1.22)	0.445

Abbreviations: Ref., reference; aIRR, adjusted incidence rate ratio; CI, confidence interval. Model adjusted for all variables included in the table.

**Table 3:** Factors associated with TB infection in asylum seekers who completed the screening.

	N. TBI/Tot (150/809)	aOR (95% CI)	*p*-Value
Gender			
Female	13/95	Ref.	
Male	137/714	1.56 (0.84–2.91)	0.163
Age at arrival, by 5 years increase		1.14 (1.01–1.29)	0.032
TB incidence in country of origin			
<150/100,0000	53/287	Ref.	
≥150/100,0000	97/522	1.02 (0.70–1.49)	0.907
Screening start			
≤6 months	111/675	Ref.	
>6 months	39/134	2.09 (1.36–3.20)	0.001

Abbreviations: Ref., reference; aOR, adjusted odds ratio; CI, confidence interval. Model adjusted for all variables included in the table.

## Data Availability

The data that support the findings of this study are available from the corresponding author upon reasonable request.
